# Influence of the patient’s age on the safety, efficacy, and prediction accuracy of the microkeratome in laser-assisted in situ keratomileusis

**DOI:** 10.1038/s41598-023-50985-6

**Published:** 2024-01-23

**Authors:** Safiya Benabidi, Andreas Frings, Vasyl Druchkiv, Toam Katz

**Affiliations:** 1 Department of Ophthalmology, Medical Faculty and University Hospital Düsseldorf – Heinrich Heine University Düsseldorf, Düsseldorf, Germany; 2 Department of Ophthalmology, University Hospital Hamburg-Eppendorf, Hamburg, Germany; 3 Clinica Baviera, Valencia, Spain; 4 Care Vision Refractive Center, Hamburg, Germany

**Keywords:** Clinical trials, Medical research

## Abstract

The purpose of this retrospective pseudonymised data analysis was to determine whether the patient’s age has an influence on the safety, efficacy, and prediction accuracy of laser in situ keratomileusis (LASIK) treatment of myopic and hyperopic eyes. This study was performed at CARE Vision GmbH (Düsseldorf, Germany) and included two patient cohorts: an older group with patients > 55 years old and a younger group with patients 30–40 years old. Each patient had a single LASIK treatment. The safety, efficacy, and prediction accuracy of the refractive results were analysed. In total, 682 patients were analysed, with 341 patients in each patient group (one eye per patient). There were 570 myopic eyes and 112 hyperopic eyes. In myopic eyes, the efficacy was significantly influenced by the patient’s age but only in myopic eyes (myopic: *p* ≤ 0.05; hyperopic: *p* = 0.085), while safety was not significantly influenced by the patient’s age in hyperopic or myopic eyes (*p* = 0.204). We found that LASIK treatment at an older age (> 55 years) resulted in almost the same safety outcomes as a LASIK treatment at a younger age (30–40 years) but with a lower efficacy; the efficacy correlated with the patient’s age. If the patient was hyperopic, their age did not influence safety or efficacy.

## Introduction

For many years, laser-assisted in situ keratomileusis (LASIK) has served as the gold standard for refractive surgery to correct a wide range of refractive errors (ametropia). Every year, there are millions of LASIK interventions throughout the world to achieve permanent correction of astigmatism, hyperopia, or myopia^1^. Here, a wafer-thin incision is made in the cornea so that a so-called ‘flap’ is created; this flap is flipped over before ablation of the corneal stroma. The tissue is removed with an excimer laser, and the flap is then folded back. As with other surgical interventions, the age of the patient at the time of the LASIK intervention has a possible influence on the refractive result^2^. For the highest possible subjective patient satisfaction after the surgery, the type and degree of the ametropia, as well as the physiological components, such as the thickness of the cornea, are of great importance. In patients with farsightedness – also known as hyperopia – the room for manoeuvring in terms of the number of dioptres is smaller than in patients with nearsightedness (myopia).

In this retrospective study, we examined the correlation between the refractive result of LASIK and the patient’s age at the time of initial laser eye treatment, with a special focus on the efficacy index (EI), the safety index (SI), and the prediction accuracy. Specifically, we examined whether the patient’s age had a negative effect on the refractive result by comparing the EI and SI between two patient groups: an older group (patients > 55 years old at the time of the intervention) and a younger group (patients 30–40 years old at the time of the intervention). López-Montemayor et al.^3^ found that in patients > 65 years old, due to lens and other age-related changes, one must deal with greater restrictions when correcting with LASIK. However, a possible causality between the patient’s age and the EI and SI has not yet been discussed. Our examination of the two age groups allowed us to make more precise and stringent statements about a possible connection between the patient’s age and the refractive result after LASIK. We also evaluated to what extent age-related degeneration of the ophthalmic apparatus could influence the effectiveness of visual acuity correction. Furthermore, based on these patient groups and parameters, we aimed to define an approximate age segment for which LASIK is particularly recommended with regard to the EI and SI.

## Methods

### Study design and patients

This study involved a retrospective pseudonymised data analysis (cross-sectional study) of patients treated consecutively at CARE Vision GmbH Germany. It included patients who had one initial LASIK treatment between January 2016 and January 2020. The older group consisted of patients > 55 years old, and the younger group included patients 30–40 years old. Only healthy patients with no other ocular pathology or systemic diseases (autoimmune diseases, cancer, etc.) and capable of giving consent were included. Patients who objected to the data analysis, with other ophthalmic diseases, or younger than 30 years at the time of the intervention were excluded. All the patients were Caucasian.

The study was approved on 7th April 2020 by the Ethics Committee of the Faculty of Medicine of the Heinrich Heine University of Düsseldorf (Study No.: 2020-1151) and was conducted according to the Declaration of Helsinki and the principles of good scientific practice. All participants provided informed consent.

### Data flow

Patient data were organised by group. Each patient in the study group was assigned a patient from the young group according to sex and initial refraction. As part of the treatment contract, each patient had already consented to participate in scientific studies and scientific pseudonymised data analysis. The groups were separated into young and old eyes. The sphere, cylinder, spherical equivalent, postoperative uncorrected distance visual acuity (UDVA, or s.c. visus = sine correctione, meaning visual acuity without correction), and preoperative corrected distance visual acuity (CDVA, or c.c. visus = cum correctione, meaning visual acuity with correction) were controlled preoperatively and postoperatively (after a 3-month follow-up).

The collected data were pseudonymised. All data were saved on CARE Vision’s internal workstations or stored in CARE Vision’s internal software (patient management software). For this study, the data were collected retrospectively and then evaluated using descriptive and deductive statistics. The pseudonymised data can be traced using a patient ID generated by CARE Vision.

### Surgical technique

The surgical procedure included a mechanical incision made in the cornea to create a ‘flap’. The excimer laser system ALLEGRETTO WAVE and the AMARIS 500 E were used to remove the tissue. A wavefront-optimised ablation profile allowed the surgeon to create large optical zones and prevent spherical aberrations. The SCHWIND AMARIS 500 E excimer laser uses a fine laser, which results in more precise and shorter treatments^4^. The Microkeratome 90 sub-Bowman by Moria was used to create the flaps.

### Statistical analysis

The EI, SI, and prediction accuracy of the refractive result were compared between the patient groups with a *t*-test. A *p*-value < 0.05 was considered to be statistically significant. To assess the influence of the patient’s age as adequately as possible, it was necessary to consider the subtle discrepancies between hyperopic and myopic eyes. For this purpose, the results were controlled by analysing subgroups (myopic and hyperopic). Of note, the older group included 7 eyes with a spherical value of 0 but a negative cylinder value. Hence, these eyes are considered to be myopic regarding the spherical equivalent and could be included in the analysis.

## Results

### Study population

**Figure 1:**
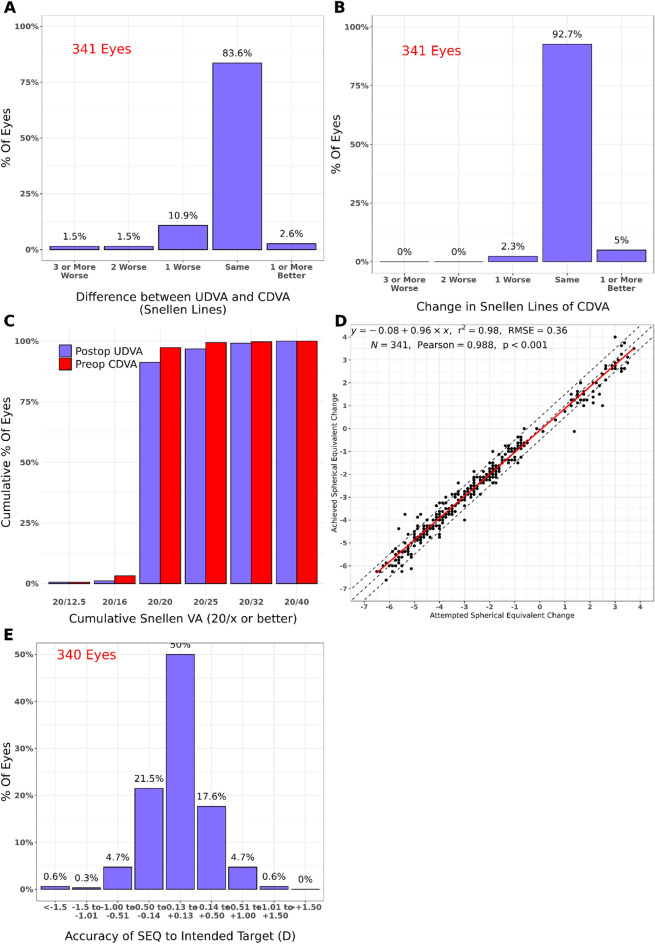
Standard graphs for reporting laser vision correction outcomes in the comparison group (patients ages 30–40 years). (**A**) Uncorrected distance visual acuity (UDVA) versus corrected distance visual acuity (CDVA); (**B**) change in CDVA; (**C**) change in UDVA; (**D**) attempted versus achieved refraction, shown as the change in spherical equivalent; (**E**) spherical equivalent refraction accuracy.

**Figure 2:**
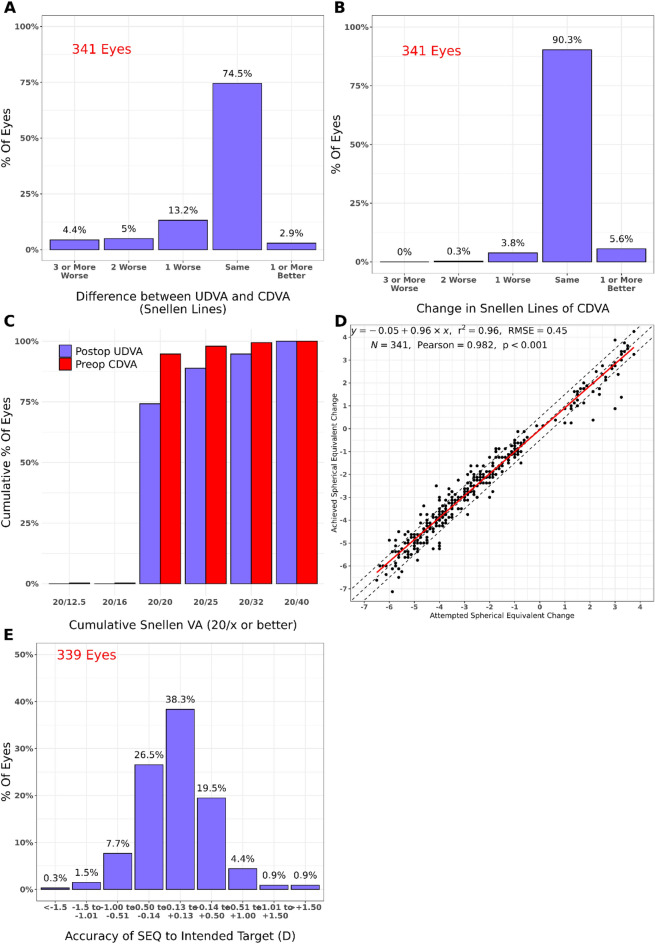
Standard graphs for reporting laser vision correction outcomes refractive outcomes in the older group (patients > 55 years old). (**A**) Uncorrected distance visual acuity (UDVA) versus corrected distance visual acuity (CDVA); (**B**) change in CDVA; (**C**) change in UDVA; (**D**) attempted versus achieved refraction, shown as the change in spherical equivalent; (**E**) spherical equivalent refraction accuracy.

After applying the inclusion criteria, the study consisted of 682 patients (570 myopic eyes and 112 hyperopic eyes; one eye per patient), with 341 eyes per group. Table 1 presents the demographic data of the patients. The median age was 35 years for the younger group and 57 years for the older group. There were no significant differences between the right and left eyes. Figures 1 and 2 present the standard graphs for reporting laser vision correction (LVC) outcomes. In the younger group, 97.1% of the eyes had a postoperative UDVA within one line or better of the preoperative CDVA. In the older group, 90.6% of the eyes had a postoperative UDVA within one line or better of the preoperative CDVA (Figs. 1A and 2A). Moreover, 2.3% of the eyes in the younger group and 4.1% of the eyes in the older group lost one or more lines of CDVA after LASIK treatment (Figs. 1B and 2B). In addition, 89.1% of the eyes in the younger group and 84.3% of the eyes in the older group had a postoperative spherical equivalent within ± 0.5 dioptres of the intended target (Figs. 1E and 2E).

**Table 1:** Demographic data of the participants.

	Younger group (N = 341)	Older group (N = 341)	Total (N = 682)	*p*-value
Sex				1.000^a^
N	341	341	682	
Female	178 (52.2%)	178 (52.2%)	356 (52.2%)	
Male	163 (47.8%)	163 (47.8%)	326 (47.8%)	
Age (years)				< 0.001^b^
N	341	341	682	
Range	30.0–40.0	55.0–75.0	30.0–75.0	
Median (Q1, Q3)	35.0 (33.0, 38.0)	57.0 (56.0, 60.0)	47.5 (35.0, 57.0)	
Eye				< 0.001^a^
OD	178 (52.2%)	237 (69.5%)	415 (60.9%)	
OI	163 (47.8%)	104 (30.5%)	267 (39.1%)	

*Q1* quartile 1, *Q3* quartile 3, *SD* standard deviation, *OD* right eye, *OI* left eye, *N* number of participants.

^a^chi-square test; ^b^Mann–Whitney test.

Table 2 summarises the refractive data. There was no significant difference in the postoperative spherical equivalent between the younger and older groups (*p* > 0.05).

**Table 2:** Preoperative, postoperative, and treatment results of younger and old patients.

	Younger group			Older group			*t*-test *p*-value
	N	Range (min to max)	Mean (± SD)	N	Range (min to max)	Mean (± SD)	
Preoperative							
Sphere (D)	341	− 6.00 to 4.00	− 1.99 (± 2.42)	341	− 6.00 to 4.00	− 1.99 (± 2.42)	1.000
Cylinder (D)	341	− 3.25 to 0.00	− 0.83 (± 0.58)	341	− 3.25 to 0.00	− 0.83 (± 0.58)	1.000
Spherical equivalent (D)	341	− 6.50 to 3.75	− 2.40 (± 2.43)	341	− 6.50 to 3.75	− 2.40 (± 2.43)	1.000
UDVA	176	− 0.20 to 2.00	1.01 (± 0.64)	165	0.00 to 2.00	1.13 (± 0.56)	0.065
DCVA	341	− 0.20 to 0.55	− 0.02 (± 0.07)	341	− 0.20 to 0.30	0.01 (± 0.06)	**0.000**
Treatment							
Sphere (D)	341	− 6.00 to 4.00	− 1.99 (± 2.42)	341	− 6.00 to 4.00	− 1.99 (± 2.42)	1.000
Cylinder (D)	341	− 3.25 to 0.00	− 0.83 (± 0.58)	341	− 3.25 to 0.00	− 0.83 (± 0.58)	1.000
Spherical equivalent (D)	341	− 6.50 to 3.75	− 2.40 (± 2.43)	341	− 6.50 to 3.75	− 2.40 (± 2.43)	1.000
Post-LASIK							
Sphere (D)	340	− 1.25 to 1.75	0.11 (± 0.40)	339	− 1.25 to 2.75	0.11 (± 0.50)	0.908
Cylinder (D)	340	− 1.50 to 0.00	− 0.26 (± 0.31)	339	− 1.75 to 0.00	− 0.32 (± 0.32)	**0.008**
Spherical equivalent (D)	340	− 1.75 to 1.50	− 0.02 (± 0.37)	339	− 1.62 to 2.12	− 0.05 (± 0.46)	0.377
UDVA	341	− 0.20 to 0.52	0.00 (± 0.09)	341	− 0.10 to 0.49	0.06 (± 0.11)	**0.000**
DCVA	341	− 0.20 to 0.52	− 0.03 (± 0.07)	341	− 0.15 to 0.30	0.01 (± 0.06)	**0.000**

*N* number of participants, *UDVA* uncorrected distance visual acuity, *CDVA* corrected distance visual acuity.

Significant values are in bold.

### Efficacy

**Figure 3:**
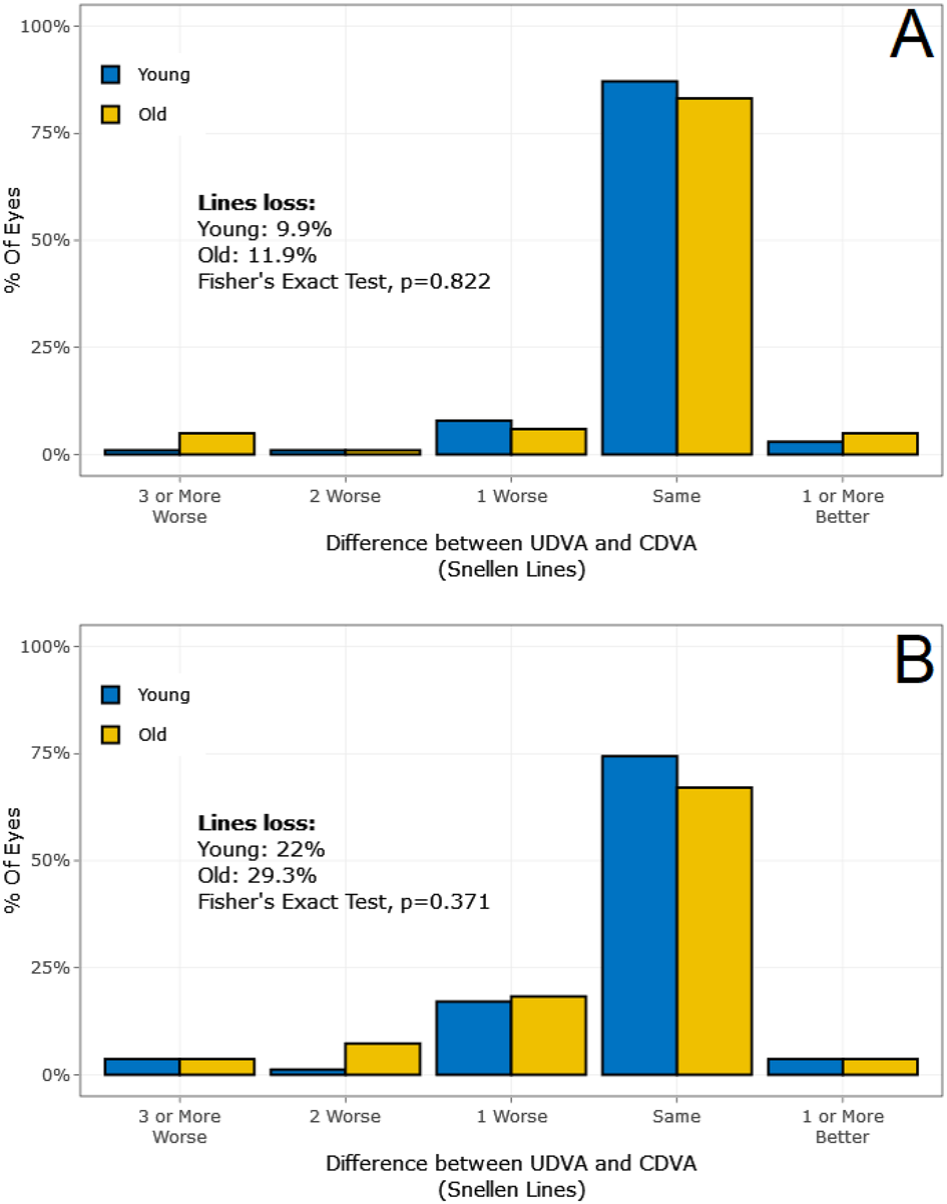
Subgroup examples. (**A**) Low myopia (0.00 to – 2.00 dioptres) and (**B**) high myopia (– 4.00 to – 6.00 dioptres).

The EI is the ratio of the postoperative UDVA to the preoperative CDVA and shows whether the patient can see after the surgery without correction as well as before the surgery with correction. The efficacy was significant in the myopic group (*t*-test, *p* < 0.05, Table 3) but not in the hyperopic group (*t*-test, *p* = 0.085, Table 4). As mentioned above, Figs. 1A and 2A show the difference between the UDVA and the CDVA (Snellen lines). We observed that 13.9% of the younger myopic patients and 22.6% of the older myopic patients lost lines after treatment. Two points are noteworthy. First, there was a higher percentage of line loss in the older group. Second, a higher percentage of line loss correlated with more severe myopia: the worse the myopia was, the more lines the patients lost (Fig. 3). We compared low (0.00 to – 2.00 dioptres) versus high myopia (− 4.00 to – 6.00 dioptres). Interestingly, compared with the myopic group, the hyperopic group lost more lines in both age groups. In sum, these results demonstrated that the efficacy was significantly influenced by the patient’s age in the myopic group (*t*-test, *p* < 0.05). Although the patient’s age did not exert a significant effect in the hyperopic group (*p* = 0.085), one can assume there was lower efficacy with older age due to the high percentage of line loss.

**Table 3:** The safety index and the efficacy index for the entire sample.

	Younger group			Older group			*t*-test *p*-value
	N	Range (min to max)	Mean (± SD)	N	Range (min to max)	Mean (± SD)	
Post-LASIK							
Safety index	341	0.75 to 1.58	1.03 (± 0.12)	341	0.56 to 1.60	1.01 (± 0.13)	0.204
Efficacy index	341	0.34 to 1.43	0.97 (± 0.16)	341	0.32 to 1.56	0.91 (± 0.19)	**0.000**

*SD* standard deviation, *N* number of participants.

Significant values are in bold.

**Table 4:** The safety index and the efficacy index for the hyperopic group.

	Young, N	Range (min to max)	Mean (± SD)	Old, N	Range (min to max)	Mean (± SD)	*t*-test *p*-value
Post-LASIK							
Safety index	56	0.75 to 1.26	1.01 (± 0.12)	56	0.73 to 1.22	0.99 (± 0.11)	0.582
Efficacy index	56	0.53 to 1.22	0.95 (± 0.15)	56	0.40 to 1.16	0.90 (± 0.19)	0.085

*SD* standard deviation, *N* number of participants.

### Safety

The SI is the ratio of the preoperative CDVA to the postoperative CDVA and represents the safety of refractive surgery. In Figs. 1B and 2B, safety is represented as the change in CDVA. The mean standard deviation of safety was 1.03 ± 0.12 in the younger group and 1.01 ± 0.13 in the older group (Table 3). In both patient groups, the SI was stable and not significantly different (*t*-test, *p* > 0.05), and pre- and postoperative SI were the statistical comparisons in young and old patients.

**Figure 4:**
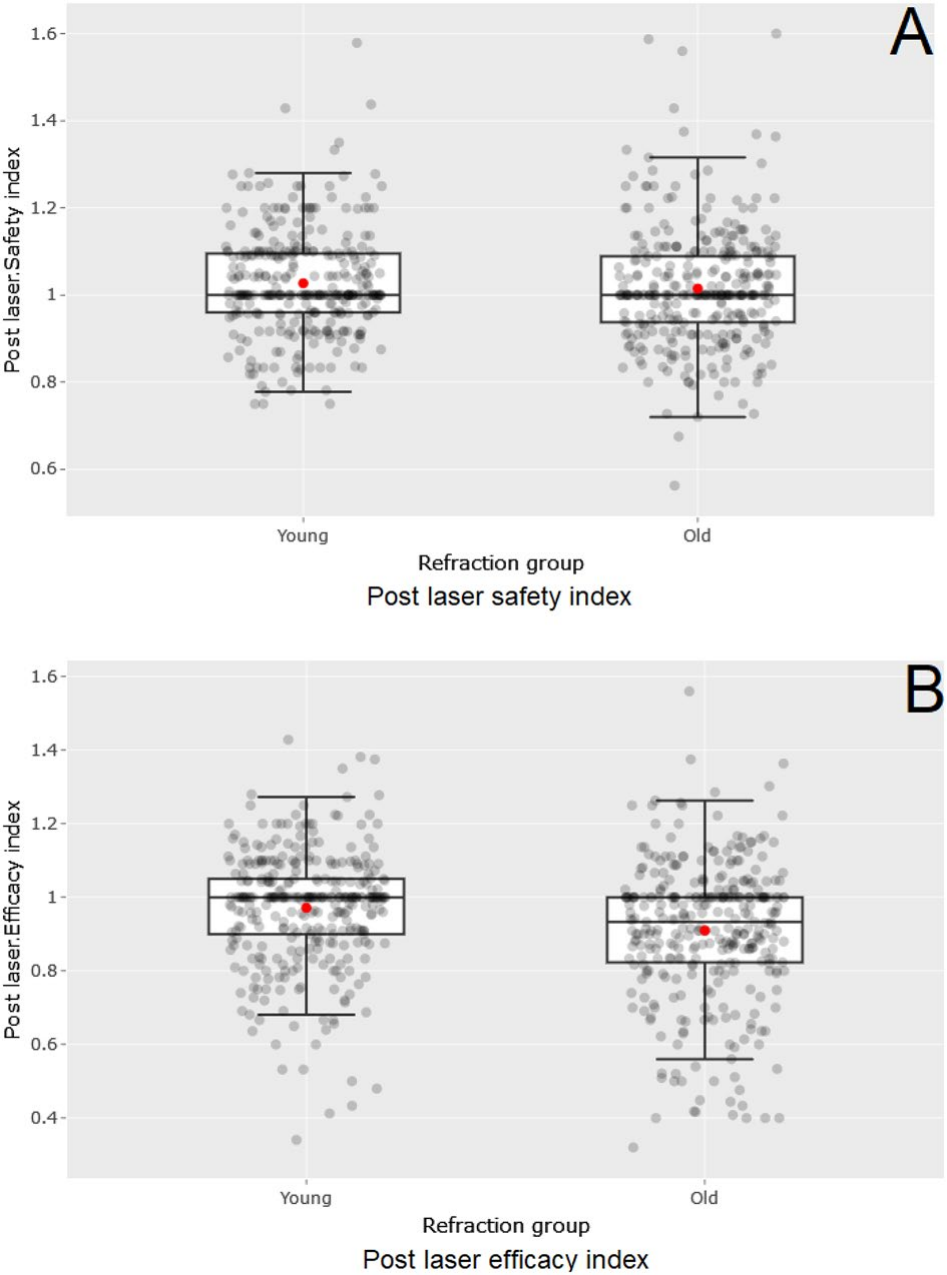
Distribution of the safety index (SI, **A**) and the efficacy index (EI, **B**). The boxplots show the distributions of the SI and EI in the specified groups. Red point = mean; black line in the box = median; grey points = outliers. The points on the line mean that the distributions are normal.

Figure 4 displays boxplots of the SI and EI and their distribution. They demonstrate the aforementioned significant changes in the EI. Note the distance of the EI from the centre line in the older group.

**Figure 5:**
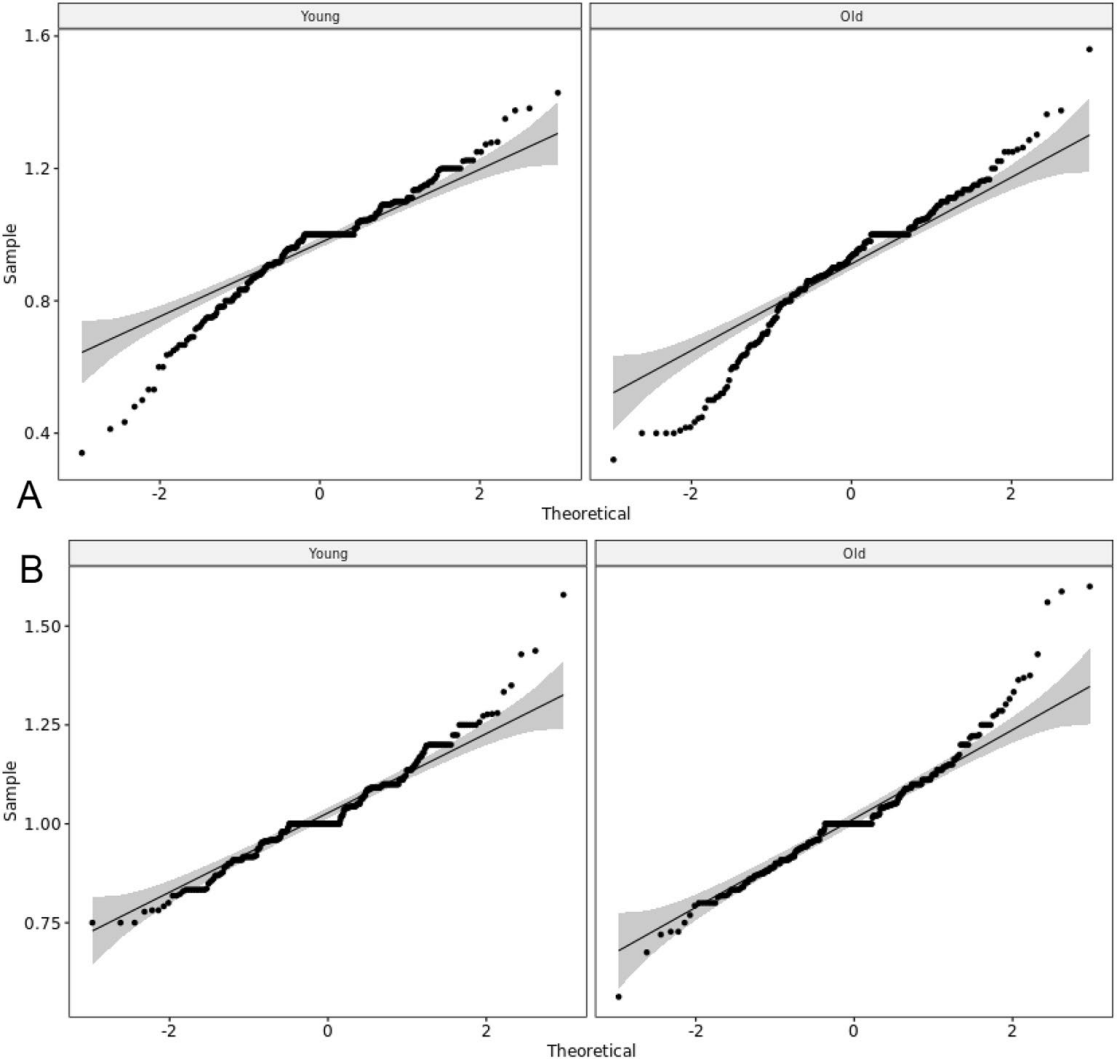
Q–Q plots showing the normality of the safety and efficacy data for the younger and older groups.

The Q–Q plots showed that the within-group distributions were normal (Fig. 5). With a large sample size, even small deviations from normality lead to a small *p*-value; hence, we used the Q–Q plots for a better visual assessment.

### Prediction accuracy

Figures 1D and 2D show the juxtaposition of the prediction accuracy after the first LASIK treatment. There was no significant difference between the attempted and the achieved spherical equivalent (*p* > 0.05, Table 2). The number of patients within the interval was higher in the younger group than in the older group. As myopia became more severe, the difference in the prediction accuracy (shown as a change in spherical equivalent) also increased. It is noteworthy that we had many more myopic patients (570 myopic vs 112 hyperopic patients). However, this phenomenon is epidemiologically justified because there are more myopic patients in the general population.

**Figure 6:**
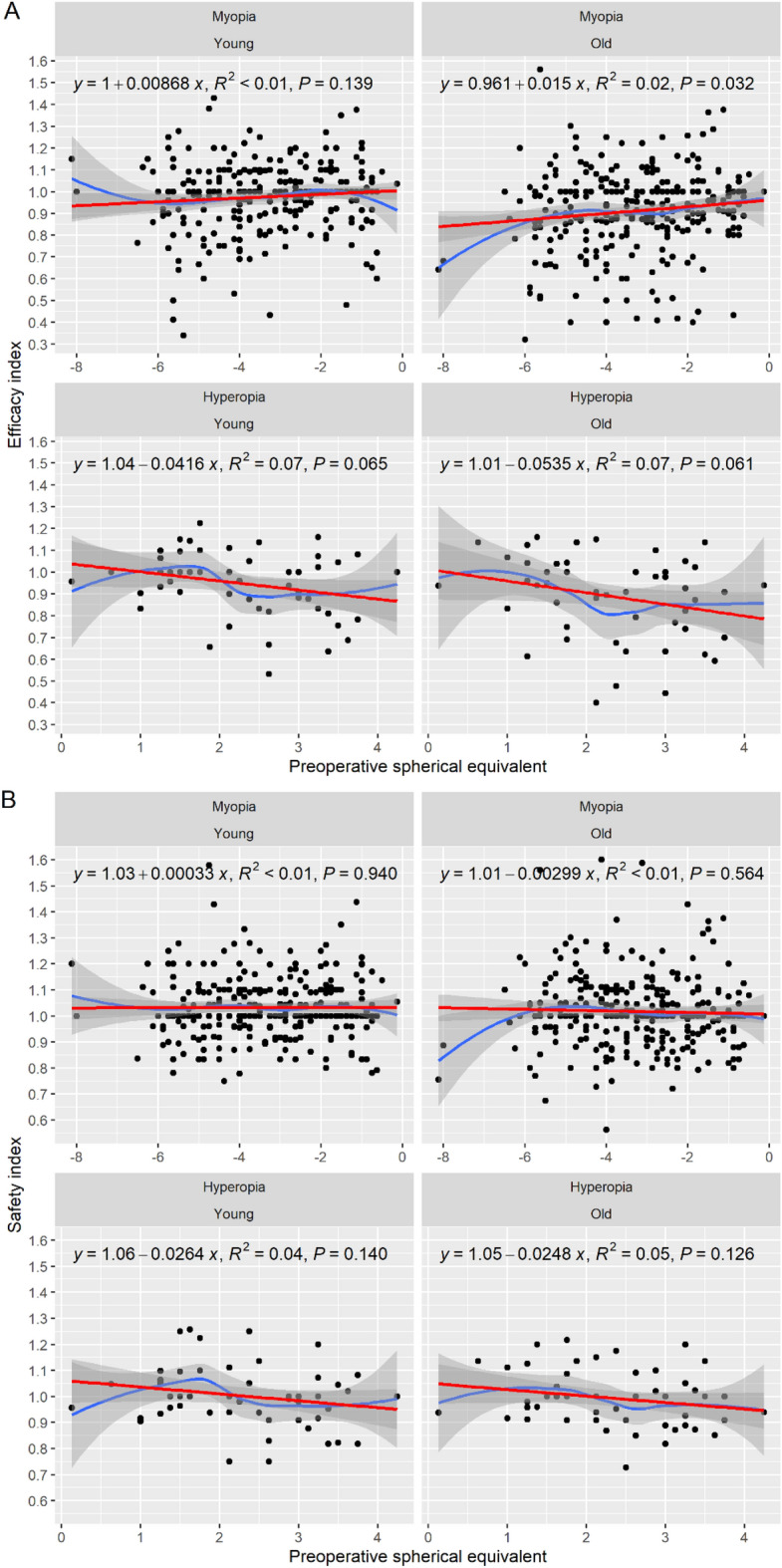
Correlation of preoperative spherical equivalent with the EI and the SI by refraction and age group.

Figure 6 shows the correlation between the preoperative spherical equivalent with the EI and SI by refraction and age group. In Fig. 6A, the blue line is based on a Loess estimation method and is useful for finding non-linear dependence. The red line is the linear regression. There was a very low but significant correlation only in the older group (*p* = 0.032). There were no other correlation patterns (the Loess curve is almost a horizontal line). When considering the age groups and their interaction with preoperative spherical equivalent as the input variables and the EI as the output variable, there were no significant effects in myopic or hyperopic eyes. There was no significant correlation between the spherical equivalent and the SI within the groups (Fig. 6B). There were no significant effects after fitting the models for myopic and hyperopic subjects with age group × preoperative spherical equivalent interaction.

## Discussion

In this study, we found comparable safety outcomes for LASIK treatment in older and younger patients but lower efficacy in older patients. A patient age > 55 years was associated with the efficacy of the surgery. In addition, a high degree of myopia correlated with a higher percentage of visual loss and, hence, lower efficacy.

López-Montemayor et al.^3^ evaluated the safety, efficacy, and refractive outcome of LASIK in patients > 65 years. Ghanem et al.^5^ assessed these variables in patients aged 40–69 years. However, despite a trend of higher retreatment rates and worse corrected visual acuity, the authors did not observe a greater risk of visual loss after LASIK treatment at an older age. Both studies found that despite an older age and the associated LASIK restrictions, the refractive outcomes and safety were satisfactory—a trend that we also observed in our study when we evaluated the results according to age. Compared with our study, the study by López-Montemayor et al.^4^ has two important limitations. First, the authors did not include a younger age group as a comparison group regarding safety, efficacy, and prediction accuracy. Second, their study group (44 eyes) had fewer cases than our study (682 eyes).

It has been shown that younger age and low myopia are indeed crucial factors and correlate with the efficacy of the treatment^6^. In that study, the researchers found that a higher SI and EI correlated with a younger age and the male sex. After evaluating our results, we could also define an age segment for the best safety and efficacy outcomes. A younger patient (30–40 years old) with low myopia (< – 2.00 dioptres) should get the best safety and efficacy outcomes. This, of course, is only a statistical and objective statement based on the results of our study. Each patient is different and will have their own personal risks and factors that might influence the safety and efficacy of surgery or any other treatment in both positive and negative ways. We did not consider factors such as temperature, season, and the surgeon’s experience; rather, we focused on the patient’s age, kind of ametropia, and the absence of ophthalmic and systemic diseases.

In summary, the patient’s age correlated with the efficacy of LASIK treatment. Furthermore, the safety was stable in both age groups. This suggests that an age > 55 years contributes to physiological components that reduce the efficacy of the surgery.

## Data Availability

The datasets generated and/or analysed during the current study are not publicly available but are available from the corresponding author on reasonable request.
